# Restless Legs Syndrome Secondary to Iron Deficiency Anaemia: A Case Report

**DOI:** 10.7759/cureus.78159

**Published:** 2025-01-28

**Authors:** Amresh Gul, Zahid Khan

**Affiliations:** 1 General Practice, General Practice Clinic, Brisbane, AUS; 2 Cardiology, University of South Wales, Pontypridd, GBR; 3 Cardiology, University of Buckingham, London, GBR; 4 Cardiology, Bart’s Heart Centre, London, GBR

**Keywords:** iron deficiency anaemia (ida), iron therapy, kidney disease, neurology case report, s: restless leg syndrome

## Abstract

Leg pain, particularly restless legs, is one of Australia's most common complaints in general practice. Iron deficiency anaemia (IDA) is a possible cause of restless legs syndrome (RLS). We present the case of a 35-year-old Aboriginal man who initially presented for general health assessment, which was required every nine months for Aboriginal and Torres Strait Islanders in Australia. He complained of restless legs at night; however, he denied any other symptoms such as tiredness and weakness. Blood tests revealed severe IDA and dyslipidaemia. After initiating iron replacement therapy, the patient’s restless leg symptoms improved significantly. This case emphasises the importance of investigating secondary causes, especially IDA. Addressing these underlying medical conditions can lead to significant improvements in patient well-being.

## Introduction

Restless legs syndrome (RLS) is a common problem encountered by patients that significantly affects their sleep and quality of life [1]. Despite a clear relationship between low peripheral iron and increased prevalence and severity of RLS, its prevalence and clinical significance in patients with iron-deficiency anaemia (IDA) are poorly understood [1,2]. RLS is primarily a clinical diagnosis following the exclusion of other causes through a thorough history and laboratory tests [3]. This condition may be idiopathic or secondary to other causes, such as iron deficiency, pregnancy, end-stage renal disease, and certain medications, such as antidepressants and antihistamines (prochlorperazine, metoclopramide, fluoxetine, sertraline and mirtazapine) [3]. Also, neuroleptic antidopaminergic, alcohol, caffeine, lithium, and beta-blockers can cause restless legs [4-6].

The pathophysiology of the RLS is not fully defined. Majority of the cases are idiopathic, in which a dysfunction of the dopaminergic system and iron stores in the brain dwindle. Perhaps, there is a tendency of autosomal dominant inheritance, which suggests the genetic basis of the condition [5,6]. In the case of uremic RLS, anaemia, especially iron deficiency, electrolyte imbalance (calcium, phosphate and potassium) and subclinical peripheral neuropathy can be the causative factors [7]. Also, polymorphisms in genes including BTBD9 and MEIS1 are linked with RLS. RLS is also common during pregnancy, especially in the third trimester [8,9].

Patients with RLS tend to develop severe pain or discomfort in their legs, prompting them to move. Patients may describe their symptoms as creeping, crawling, itchy, burning, searing, tugging, pulling, aching, hot or cold, electric, restless, or painful. Although it is uncommon for symptoms to be limited solely to the arms, approximately half of the cases involve both arms and legs [10]. Symptoms mainly occur after the patient has been lying still and can last for several minutes to an hour [10]. However, symptoms can also arise whilst sitting quietly, and this occurrence is less frequent [10,11]. Furthermore, the severity of symptoms is often aggravated when the patient is mentally relaxed and physically inactive. Symptoms are usually more prominent from evening until early morning, regardless of the patient's sleep state. In severe cases, this circadian rhythm may be disrupted [12,13]. Factors such as shift work, specific medications, and sleep disorders can also affect the pattern of symptoms [13]. Most cases of RLS are mild and typically do not require treatment or intervention.

For those experiencing mild symptoms, lifestyle changes including regular physical exercise, good sleep practices and dietary changes are recommended [7]. It is also imperative to rule out iron deficiency, as it can cause and worsen RLS [7]. Iron supplements should be administered to achieve a serum ferritin concentration of at least 50 μg/L if deficiency is present [12,13]. The recommended dose of oral therapy ferrous fumarate is 200 mg (67.5 mg iron) twice a day [13].

## Case presentation

A 35-year-old Aboriginal man came to our general practice clinic with his partner (wife) for a health problem to be addressed, which was dealt with accordingly. During the Aboriginal health assessment as per the Australian guidelines for Aboriginal and Torres Strait Islanders, he further added that he had been enduring leg discomfort, which he delineated as restless for the last few months, and it was the first time he experienced it. The onset of the restlessness was sudden and gradually increased. Further, he added that it mainly happens at nighttime, and his sleep gets broken, eventually affecting his daily routine. In the morning, he was not feeling fresh and experienced tiredness. 

His wife also noticed that he had been moving his legs prodigiously whilst sleeping, which concerned her about his health. He denied symptoms of tiredness, weakness, shortness of breathing, gastrointestinal symptoms and loss of appetite. He also denied the symptoms of obstructive sleep apnoea. On diet history, he was not consuming red meat, and his fruit intake was minimal. There was no significant family history related to IDA or gastrointestinal diseases; however, there was a considerable history of diabetes and heart conditions.

On examination, his blood pressure was 114/72, his heart rate was 82 beats per minute, and his BMI was 26.5. The rest of the examinations, including the neurological examination, were unremarkable. He was educated about the possibilities of his leg discomfort's secondary causes, which can possibly cause his restlessness of the legs. On the follow-up visit, his blood workup showed that his serum ferritin of 10 ug/L and haemoglobin of 121g/dl were low (Table 1). He also had moderate dyslipidaemias. Regarding his elevated liver function enzymes, his ultrasound confirmed a fatty liver. His coeliac antibodies were negative, and vitamin B12 was normal.

Consequently, he was started on oral iron replacement therapy (ferrous fumarate 200 mg, 967.5 mg iron) twice a day, and advised lifestyle modification focused on consuming more red meat, fruits, and vegetables. On a follow-up visit after three months, his symptoms were substantially improved, and his sleep improved. His ferritin level was increased to 26 ug/L on the follow-up visit. He was advised to continue the medicine along with the aforementioned dietary modification and recommended to make follow-up visits.

**Table 1:** The laboratory test results confirmed severe iron deficiency anaemia MCV: mean corpuscular volume; TSH: thyroid-stimulating hormone; TIBC: total iron-binding capacity; eGFR: estimated glomerular filtration rate; ASP: alkaline phosphatase; AST: aspartate aminotransferase; ALT: alanine transaminase; GGT:  gamma-glutamyl transferase; HDL: high-density lipoprotein; LDL: low-density lipoprotein

Lab test	First visit	Three months follow-up	Six months follow-up	Reference range
Haemoglobin	137 g/l	121 g/l	140 g/l	135-175 g/L
MCV	78 g/l	80 g/l	82 g/l	80-100 g/L
Platelets	331 10*9L	354 10*9L	350 10*9L	150-400 10*9L
Haematocrit	0.40	0.39 mIU/L	-	0.40-0.54
TSH	2.5 mIU/L	2.5 mIU/L	-	0.3-3.5 mIU/L
Iron	9 umol/L	7 umol/L	15 umol/L	5-30 umol/L
Transferrin		4.0 g/l	3.8 g/l	1.9-3.1g/L
TIBC		101 umol/L	90 umol/L	47-77 umol/L
Ferritin		10 ug/L	26 ug/L	30-300 ug/L
Trans saturation		7 %	20%	20-45%
Sodium	137 mmol/L	137 mmol/L	-	135-145 mmol/L
Potassium	4.4 mmol/L	4.3 mmol/L	-	3.5-5.5 mmol/L
Creatinine	93 umol/L	105 umol/L	-	60-110 umol/L
eGFR	90	79	-	>60
Albumin	46 g/L	41 g/L	-	35-48g/L
Total bilirubin	16 umol/L	6 umol/L	-	<21 umol/L
ALP	105 U/L	97 U/L	-	35-110 U/L
AST	30 U/L	32 U/L	-	10-40 U/L
ALT	54 U/L	70 U/L	-	5-40 U/L
GGT	87 U/L	106 U/L	-	5-40 U/L
Total cholesterol	5.4 mmol/L	6.2 mmol/L	-	<5.6 mmol/L
Triglycerides	-	5.3	-	<2.1 mmol/L
HDL	-	0.92	-	>0.89 mmol/L
LDL	-	3.1	-	<4.1 mmol/L

## Discussion

RLS, a sensorimotor disorder affecting the nervous system, has a prevalence rate of 10% [14]. Notably, this condition is particularly prevalent among individuals with end-stage renal disease [7]. RLS is characterised by an involuntary and intolerable sensation of pain or unusual feelings, such as crawling or burning sensations, predominantly occurring in the lower extremities [11-13]. These symptoms often manifest or intensify during periods of rest and at night [14,15].

The pathogenesis of RLS is complex and constantly evolving with new research. It involves a myriad of factors, including deficiencies in brain iron, disruptions in dopaminergic neuronal transmission, peripheral neuropathy, chronic inflammation and immune system deficiencies [16]. Recent research has indicated that the onset of RLS is linked to heightened activation of sympathetic nerves, with inflammation and immune alterations frequently observed in individuals affected by RLS [17]. It is also precipitated by multiple medications such as antidepressants and antihistamines (prochlorperazine, metoclopramide, fluoxetine, sertraline and mirtazapine [3,9,18].

An effective strategy for managing RLS/Willis-Ekbom disease (WED) involves a precise diagnosis, a crucial step that underscores the importance of healthcare providers in patient care [13]. Recognising reversible contributing factors and implementing nonpharmacological interventions, such as iron supplementation (either orally or intravenously), are also key [18,19]. Numerous pharmacological options are available for the treatment of RLS/WED. Until recently, the primary treatment approach for RLS/WED has been the administration of low-dose dopamine agonists (DAs) [13,18].

Iron deficiency affects 25% of RLS individuals. A double-masked trial showed that oral iron therapy provided symptomatic relief for RLS patients with low to normal serum ferritin levels (15-75 ng/ml). Ferrous sulphate at 325 mg daily or ferrous fumarate at 200 mg (67.5 mg iron) twice a day is recommended for those with ferritin below 75 ng/ml, ideally taken with vitamin C to enhance absorption [20]. Some patients may require intravenous iron due to oral intolerance or unresponsive idiopathic RLS, though this method carries a risk of anaphylaxis and should be used cautiously [21].

## Conclusions

RLS poses challenges for healthcare providers regarding diagnosis and treatment due to our incomplete comprehension of the underlying pathophysiological mechanisms associated with these conditions. Patients should be advised to avoid trigger medications which cause the symptoms. In case of chronic kidney disease patients or dialysis, regular monitoring, correction of electrolyte imbalance corrections and vitamin D supplements in case of deficiency are advisable. Numerous treatments are available for RLS, but healthcare providers must exercise utmost caution to prevent the augmentation that frequently arises with dopaminergic therapies. DAs, including pramipexole, ropinirole, rotigotine and cabergoline, have been found to alleviate symptoms and increase sleep quality and overall quality of life. Nevertheless, pramipexole and ropinirole do cause their own adverse effects, such as the risk of gambling addiction and significant weight gain. On the other hand, the rotigotine transdermal patch is a well-tolerated option, with a relatively low risk of causing clinically significant augmentation of RLS. It is imperative to consider underlying causes of restless legs before making a final diagnosis, such as IDA, kidney failure, medication-induced electrolyte imbalance and pregnancy. This responsibility underscores the weight of our role in patient care.
